# Efficacy of a Botanical Supplement with Concentrated *Echinacea purpurea* for Increasing Aerobic Capacity

**DOI:** 10.1155/2014/149549

**Published:** 2014-01-16

**Authors:** David Bellar, Kaitlyn M. Moody, Nicholas S. Richard, Lawrence W. Judge

**Affiliations:** ^1^School of Kinesiology, University of Louisiana at Lafayette, 225 Cajundome Blvd, Lafayette, LA 70506, USA; ^2^School of Physical Education, Sport & Exercise Science, Ball State University, 2000 W. University Ave, Muncie, IN 47306, USA

## Abstract

The present investigation evaluated the efficacy of a botanical supplement that delivered a concentrated dose of *Echinacea purpurea* (8 grams day^−1^). The participants were 13 apparently healthy, recreationally active college students (VO_2_ max: 51 mL O_2_/kg∗min). The participants were provided with a 30-day supplementation regime. Data regarding maximum aerobic capacity was collected through pre- and posttesting surrounding the 30-day supplementation regime. The participants were instructed to maintain normal levels of physical activity and exercise during the experimental period. The levels of physical activity and exercise were monitored via the Leisure and Physical Activity Survey. The participants did not report any significant increases in aerobic physical activity or exercise during the supplementation period. Paired samples *t*-test analysis did not reveal a significant difference in maximum aerobic capacity, *t*(12) = 0.67, *P* = .516. Presupplementation maximum aerobic capacity (*M* = 51.0, SD = 6.8) was similar to postsupplementation values (*M* = 51.8, SD = 6.5). This study suggests that botanical supplements containing a concentrated dose of *Echinacea purpurea* is not an effective intervention to increase aerobic capacity of recreationally active individuals.

## 1. Introduction

Maximal oxygen consumption (VO_2_ max) is the amount of oxygen the human body can utilize in muscles and/or tissues. VO_2_ max testing is the laboratory standard for assessing aerobic fitness [1]. Low aerobic fitness is associated with congestive heart failure, anemia, and obstructive pulmonary disease [2]. VO_2_ max is determined by both the capacity of oxygen delivery to the blood stream and the capacity of oxygen extraction from blood [3].

Botanical supplements have been studied for decades, some of which have been associated with health or performance benefits [4]. Echinacea is an herbal supplement that is derived from the North American Purple Coneflower plant and has shown some potential mechanisms that, with supplementation, could augment oxygen transport. Echinacea supplementation was found to increase VO_2_ max likely through an increase in the number and size of red blood cells, hemoglobin, and hematocrit associated with increases in serum erythropoietin [1]. Recent research suggests Echinacea induced erythrocythemia and VO_2_ max results in an increase in serum erythropoietin (EPO) [1, 5]. This glycoprotein hormone, primarily produced in the kidneys, regulates red blood cell production. EPO improves exercise performance by increasing oxygen blood transport, which results in a greater VO_2_ max [6]. The increase in red blood cell mass is proceeded by a decrease in plasma volume to control hemoconcentration [7]. The regulation of EPO production is based upon oxygen concentration in the blood and is increased by any condition that results in a decrease in the quantity of oxygen that is transported in the blood to the tissues [1, 8].

Running economy (RE) is the energy cost reduction of submaximal running evaluated through the measurement of oxygen consumption during steady state exercise [8, 9]. Potential mechanisms for improved submaximal RE ensuing from erythrocythemia include an increase in the amount of adenosine triphosphate (ATP) produced per mole of oxygen consumed, a decrease in the amount of ATP necessary for running at a given speed, or a combination of both mechanisms [1, 8, 9]. Another adaptation resulting from erythrocythemia is a reduction in the heart rate [8, 9]. Based upon the literature, it is reasonable to assume that Echinacea will increase EPO and, in turn, affect erythrocyte production. However, oxygen availability is generally not a limiting factor for exercise in normobaric environments [7]. Therefore, an increase in EPO alone may not be sufficient to increase exercise performance or maximum aerobic capacity, where both delivery and extraction of oxygen by tissue are necessary to increase VO_2_ max.

Dietary supplements are a growing trend in the United States [10]. The numbers of ergogenic aids available to athletes are steadily rising [11–13]. Recent studies have shown an increase in supplementation usage, which may be related to the growing competitiveness in sports on both the collegiate and professional level [14–16]. In a study of male and female elite figure skaters, supplement use was reported to prevent illness and disease, to increase energy, and to enhance performance [17]. Echinacea is readily available in the United States in multiple forms. Given the available information regarding Echinacea, it is important to evaluate whether or not supplementation is effective at increasing maximum aerobic capacity in the absence of an increased volume of physical activity and exercise.

The purpose of this study was to determine the effects of oral Echinacea supplementation on VO_2_ max and economy during submaximal treadmill walking and running in human participants. It was hypothesized that Echinacea supplementation might augment running performance in humans based upon previous evidence of changes in EPO hormones levels demonstrated in previous research [1, 5].

## 2. Materials and Methods

### 2.1. Experimental Design

The present investigation involved a controlled pre-/postdesign for physical activity. The local Institutional Review Board approved the study for the use of human subjects. All subjects provided informed written consent prior to beginning the study and were aware of their right to withdraw at any time. Participants were tested for maximum aerobic capacity, provided with a 30-day supply of supplements, and were asked to stringently adhere to their typical routine and levels of physical activity and exercise. Following the completion of the 30-day supplementation period, each participant was again tested for maximum aerobic capacity. All subjects reported consumption of all doses of the required supplement.

### 2.2. Participants

Participants for the present investigation were 13 apparently healthy college age males. The subjects were recruited based upon being recreationally active without being a competitive athlete. Participant information can be seen in Table 1.

### 2.3. Botanical Supplement

The botanical supplement (VO_2_- Advantage, Second Wind LLC, Highland Park, IL, USA) contained only substances that are generally regarded as safe by the United States Food and Drug Administration. The product contains a concentrated dose of *Echinacea purpurea* (8 grams ∗ day^−1^), as well as *Rhodiola rosea*, *cordyceps sinesis*, quercetin, beta alanine, catechins, vitamin C, vitamin B-3, vitamin B-12, iron, folic acid, inositol, and alpha-lipoic acid. The supplement is taken in 4 daily doses via ingestible capsules. The subjects were provided with a 30-day supply of the supplement, and all subjects were compliant in consuming the required doses.

### 2.4. Maximum Aerobic Capacity Assessment

The graded exercise test format included the use of the same equipment during both pre- and posttesting sessions. Participants utilized the same equipment in the Human Performance Lab. The subjects ran on a Track Master TMX 425 treadmill (Full Vision Inc., Newton, KS.) during the assessment. Participant's expired air was sampled and analyzed with a ParvoMedic TrueOne 2400 metabolic measurement system (ParvoMedics, Sandy, UT). The system utilized a mixing chamber and was set to report data every 10 seconds. The system was calibrated prior to each test according to the manufacturer's specifications. Drift in the sensors was not appreciable during testing. Listed accuracy for the gas sensors in the unit are paramagnetic O_2_ analyzer ±0.1%, infrared CO_2_ analyzer ±0.1%, and pneumotach ±2%.

The Bruce Protocol was used for the assessment [6]. The test was concluded when the oxygen consumption was determined to have reached a plateau, or the participant volitionally ceased exercise. Participants were determined to have reached VO_2_ max if they demonstrated a plateau in oxygen consumption (<100 mL O_2_ change with increasing workload) or achieved an RER value of greater than 1.15. Heart rate during the test was determined through a Polar Wear Link heart rate sensor (Polar Electro Inc., Lake Success, NY) that was linked to a receptor on the metabolic measurement system.

### 2.5. Leisure and Physical Activity Survey

The LPA is a short self-report instrument designed to assess college student's sedentary/nonsedentary activity, class rank, gender, and grade point average. Demographic variables are recorded as follows: class rank as freshman, sophomore, junior, or senior, gender as female or male, and grade point average between 0.0 and 4.0. Types of sedentary activity include time spent in typing/schoolwork, web surfing/entertainment, and video gaming. Each sedentary/nonsedentary activity type classification has further descriptors for clarification: web surfing/entertainment included (television, facebook, myspace, etc.) and video gaming included (XBox, XBox 360, Playstation, etc.). These activities were assessed for frequency (0–2 days, 3–5 days, and 6-7days) per week as well as duration per bout (0–15 min, 16–30 min, and greater than 30 min). Each frequency and duration was assigned a score of 1 to 3 pts for each of the possible responses. Grade point average was assessed via predetermined ranges of answers (0–0.99, 1–1.99, 2–2.99, 3–3.99, 4.0, or above). Aerobic exercise (running, walking, biking, aerobics classes, etc.) and weightlifting (machine, free weight, etc.) were assessed in a similar fashion for frequency and duration.

Total scores for each item assessed were computed as the sum of the frequency and duration scores. The instrument has demonstrated test-retest reliability and criterion referenced validity [18]. In order to determine if an increase in physical activity and exercise duration was present, participants were assessed at the beginning and at the end of the supplementation period through the use of the LPA survey (Table 2).

### 2.6. Statistical Analysis

Prior to analysis, all continuous variables were checked for normality using Shapiro-Wilks analysis. Paired samples *t*-test was also used to evaluate pre- and posttests of maximum aerobic capacity. Wilcoxon Signed Ranks tests were used to compare activity levels reported on the LPA survey from the start of the study to the conclusion of the study. A repeated measures ANOVA (Pre-Post Supplementation ∗ Stage of Graded Exercise Test) was used to evaluate changes in economy of movement during the assessment of maximum aerobic capacity. All analyses were performed with the use of a modern statistics software package (SPSS ver 21.0, IBM, New York, USA).

## 3. Results

### 3.1. Pre- to Post- LPA Survey

The data collected for the survey was ordinal in nature; therefore nonparametric procedures were employed for analysis. The results of the Wilcoxon Signed Ranks test suggest that there were no significant changes in aerobic activity level, *Z*(12) = 1.577, *P* = .115 nor were there significant changes in weight lifting activity, *Z*(12) = 1.826, *P* = .068. Based upon the lack of significant change in reported activity, maximum aerobic capacity before and after was analyzed independent of self-reported activity levels.

### 3.2. Pre- to Postmaximum Aerobic Capacity

**Figure 1:**
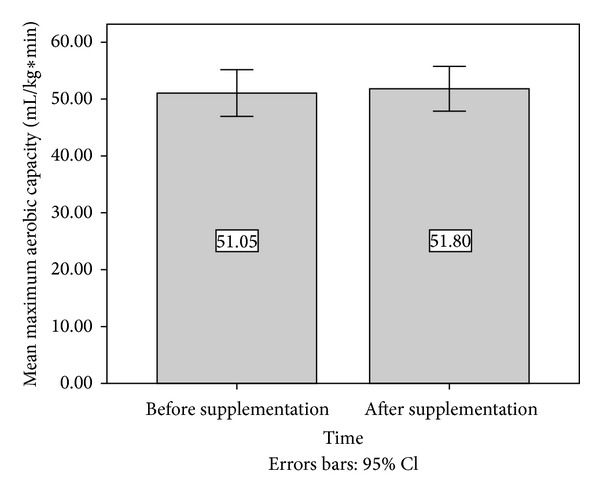
Pre- and postsupplementation maximum aerobic capacity (mL O_2_/kg ∗ min). Error bars represent 95% confidence intervals.

Data collected from graded exercise tests to determine maximum aerobic capacity were found to be normally distributed via Shapiro-Wilks analysis (*P* > .45). Therefore, paired samples *t* test analysis was undertaken. Paired samples *t*-test analysis did not reveal a significant difference in maximum aerobic capacity, *t*(12) = 0.67, *P* = .516. Presupplementation maximum aerobic capacity (*M* = 51.0,  SD = 6.8) was similar to postsupplementation values (*M* = 51.8,  SD = 6.5).  Figure 1 summarizes the findings in regard to maximum aerobic capacity.

### 3.3. Economy during Graded Exercise Test

**Figure 2:**
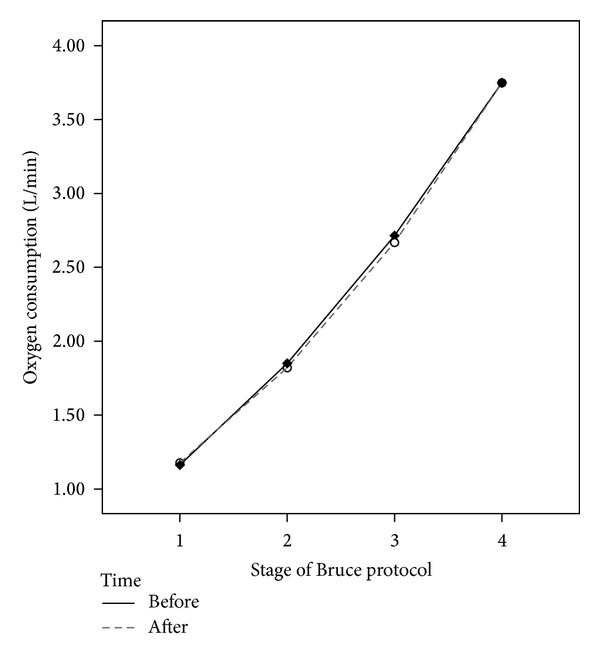
Walking and running economy (L O_2_/min) for 4 stages of the graded exercise test before and after supplementation.

The first four stages of the Bruce protocol were analyzed. For each stage, the 3 minutes of absolute oxygen consumption (L O_2_/min) was averaged for the comparison. The repeated measures ANOVA did not reveal a main effect for changes in economy pre to post, *F*(1,11) = 0.067, *P* = .800, nor an interaction effect for time (pre, post) by stage (1–4), *F*(3,33) = 0.669, *P* = .577.  Figure 2 summarizes the findings in regard to exercise economy.

## 4. Discussion

Based upon the present investigation, a botanical supplement with a concentrated dose of Echinacea is not sufficient to increase maximum aerobic capacity. This finding is in contrast to previous reports in the literature that reported increases in VO_2_ max with Echinacea supplementation [1]. In that study, a similar dose of 8 grams per day of Echinacea was administered for 28 days (the present study used 30 days), and significant increases in VO_2_ max in the supplement group were noted. However, the increase in maximum aerobic capacity in the supplement group was very small, indicated by an increase of only 1.5%. This result is likely to be statistically significant but the practical significance is questionable given the very small effect of the supplement. A more recent study [19] is in agreement with the present study which found that an 8 gram per day dose of Echinacea was not found to increase the maximum aerobic capacity of trained distance runners. It may be important to note that all three studies utilized a similar does of *Echinacea Purpurea*, which allows for comparison of the effects. In the present investigation and the Baumann et al. [19] study, there was almost no change in the mean aerobic capacity of the participants who received the supplement. In the Whitehead et al. [1] study, there was a minimal change in aerobic capacity. The duration of supplementation was variable among the studies from 3 to 6 weeks. However, the longest use of the supplementations resulted in no difference in VO_2_ max [19]. In regard to economy, the present investigation did not reveal any changes in walking or running economy during the first 4 stages of the graded exercise test. This is, again, in contrast to the changes reported by Whitehead et al. [1] but is similar to the reported changes in VO_2_ max. This study reported significant, but very minimal, changes in walking/running economy during the first 2 stages of a graded exercise test (Stage 1: 1.5% increase in economy; Stage 2: 1.67% increase in economy). Based upon the available evidence, it seems unlikely that Echinacea supplementation has a meaningful impact on exercise economy during walking and running.

The limitations of the present study involved the use of self-reported compliance for adherence to the supplementation regime, the use of a self-reported instrument (LPA survey) to examine potential changes in physical activity and exercise behavior, and the use of supplement that had components other than Echinacea. Another limitation was the lack of a control group or placebo treatment. Though these limitations are necessary to disclose, the nature of the study was likely similar to the use of the supplement in a real-world setting. Therefore, the results are applicable to those who have an interest in exercise or sport nutrition.

## 5. Conclusions

Based upon the present study, the use of a botanical supplement with a concentrated dose of *Echinacea Purpurea* cannot be recommended to increase aerobic capacity or economy during exercise in healthy, recreationally active adults. Given the information regarding the ability of Echinacea to augment EPO levels, these supplements should be evaluated for use in populations where oxygen delivery limits functional capacity, such as those individuals with disease states or those persons who will transition to living at altitude.

## Figures and Tables

**Table 1:** Participant characteristics.

	Mean	SD
Age (yrs)	25.6	3.7
Height (cm)	177.3	7.1
Weight (kg)	82.8	11.8
Maximum aerobic capacity (mL/kg∗min)	51.7	7.0

Participant characteristics: means ± SD. Participants are *n* = 13 males.

**Table 2:** LPA survey results for aerobic activity.

	Mean	SD
Presupplement days of aerobic exercise	1.8	0.7
Postsupplement days of aerobic exercise	1.6	0.7
Presupplement duration of aerobic exercise	2.2	0.7
Postsupplement duration of aerobic exercise	1.9	0.8

Aerobic scores from LPA: means ± SD. These activities were assessed for frequency (1 = 0–2 days, 2 = 3–5 days, and 3 = 6-7 days) per week as well as duration per bout (1 = 0–15 min, 2 = 16–30 min, and 3 = greater than 30 min).
